# Apathy, but not depression, is associated with executive dysfunction in cerebral small vessel disease

**DOI:** 10.1371/journal.pone.0176943

**Published:** 2017-05-11

**Authors:** Valerie Lohner, Rebecca L. Brookes, Matthew J. Hollocks, Robin G. Morris, Hugh S. Markus

**Affiliations:** 1 Stroke Research Group, Department of Clinical Neurosciences, University of Cambridge, Cambridge, United Kingdom; 2 Department of Psychology, Kings’ College London, Institute of Psychiatry, Psychology and Neuroscience, London, United Kingdom; Charite Universitatsmedizin Berlin, GERMANY

## Abstract

**Objective:**

To determine the prevalence of apathy and depression in cerebral small vessel disease (SVD), and the relationships between both apathy and depression with cognition. To examine whether apathy is specifically related to impairment in executive functioning and processing speed.

**Methods:**

196 patients with a clinical lacunar stroke and an anatomically corresponding lacunar infarct on MRI were compared to 300 stroke-free controls. Apathy and depression were measured using the Geriatric Depression Scale, and cognitive functioning was assessed using an SVD cognitive screening tool, the Brief Memory and Executive Test, which measures executive functioning/processing speed and memory/orientation. Path analysis and binary logistic regression were used to assess the relation between apathy, depression and cognitive impairment.

**Results:**

31 participants with SVD (15.8%) met criteria for apathy only, 23 (11.8%) for both apathy and depression, and 2 (1.0%) for depression only. In the SVD group the presence of apathy was related to global cognition, and specifically to impaired executive functioning/processing speed, but not memory/orientation. The presence of depression was not related to global cognition, impaired executive functioning/processing speed or memory/orientation.

**Conclusions:**

Apathy is a common feature of SVD and is associated with impaired executive functioning/processing speed suggesting the two may share biological mechanisms. Screening for apathy should be considered in SVD, and further work is required to develop and evaluate effective apathy treatment or management in SVD.

## Introduction

Cerebral Small Vessel Disease (SVD) is the most common cause of vascular cognitive impairment and vascular dementia, with up to 50% of patients with lacunar stroke having some degree of cognitive impairment [1, 2]. These impairments characteristically involve cognitive flexibility, attention, and processing speed [3–5], with episodic memory relatively spared [6, 7]. Neuropsychiatric comorbidities, particularly depressive symptoms [8–10] are increasingly being linked to SVD. Some of the scales used to measure depression include items relating to both apathy and depression. Recently apathy, rather than depression, has been suggested as the major neuropsychiatric symptom in this group [11] and in patients with dementia with co-existing WMH [12]. For example, in one recent study in patients with clinical lacunar stroke and confluent WMH, apathy, but not depression, was associated with the extent of white matter damage, estimated using diffusion tensor imaging (DTI) [11]. Following from this paper, it is important to replicate the finding that apathy is an important neuropsychiatric symptom in further SVD cohorts, and to determine whether this association is seen across patients with a wide variety of SVD severity; in this paper all cases had lacunar infarcts as well as confluent white matter hyperintensities, indicating more severe disease.

Apathy is a syndrome consisting of behavioral, affective, and cognitive features [13]. It is commonly considered to be an intrinsic component of cognitive decline [14] with diminished motivation, initiative and interest, and blunting of emotions as core features [15]. Similarly, apathy is often considered to be a component of the ‘dysexecutive syndrome’, which can occur as a result of acquired brain injury or dysfunction involving the fronto-subcortical circuits [16, 17]. This is supported by studies of both Alzheimer’s and Parkinson’s disease [18, 19]. To the best of our knowledge, studies on the relationship between apathy and impaired cognition in SVD are currently lacking and hence this needs to be addressed.

In this study we determined firstly the prevalence of both apathy and depression in a population with symptomatic lacunar stroke in comparison to a healthy control group. Secondly, in the SVD group we investigated the relationship between both apathy and depression with cognition, and determined whether apathy is specifically related to executive functioning and processing speed.

## Materials and methods

### Standard protocol approvals, registrations, and patient consents

All participants signed an informed consent form before taking part in this study. The London Bridge Research Ethics Committee approved this study (11/LO/0636).

### Study population

Two-hundred participants with SVD (age range 35 to 100 years) were recruited from stroke wards and outpatients clinics at 19 sites across the English Stroke Research Network, between July 2011 and September 2013, as part of a multicenter study to validate a tailored SVD cognitive screening tool (see below for a list of all sites). The sample size was calculated based on a meta-analysis examining studies investigating the relationship between WMH and cognition; taking into account that the sample of 200 participants should lead to acceptable significance levels for a range of different analyses, including additional correction for multiple comparisons [20]. SVD was defined as having a symptomatic lacunar syndrome [21] with MRI confirmation of an anatomically relevant infarct on MRI. For MRI in the acute phase this was an acute infarct on diffusion-weighted imaging, and for non-acute MRIs an anatomically relevant lacunar infarct visible on FLAIR or T1 sequences. Exclusion criteria included any stroke subtype than SVD; this included stenosis >50% in the extracranial or intracranial cerebral vessels, or previous carotid endarterectomy, cardioembolic source of stroke, defined according to the TOAST criteria [22] as high or moderate probability, and/or the presence of a cortical infarct >1cm diameter on MRI. Further exclusion criteria were clinical dementia, and lack of sufficient fluency in English to allow cognitive testing. All participants were tested at least three months post-stroke to reduce any acute effects of stroke on cognitive performance. Participant MRI scans were centrally reviewed to confirm eligibility.

Patients with lacunar stroke with or without WMH were included. However, the degree of WMH was assessed on central blinded review using the semi-quantitative Fazekas’ scale [23]. The SVD cohort was then categorized into those with either lacunar infarcts without WMH (Fazekas scale 0 or 1; n = 122), or lacunar infarcts with confluent WMH (Fazekas ≥2; n = 74).

Three-hundred healthy controls were recruited from local family doctors practices or other volunteer groups in South London. They were matched on socioeconomic status and years of education, and were included as a reference group for cognitive, apathy and depression scores. Individuals with cardiovascular risk factors and other comorbidities were included, but individuals with a past history of stroke, transient ischemic attacks, and other major central neurological or major psychiatric diseases were excluded [20]. Brain imaging was not performed in the controls.

For both groups hypertension, hypercholesterolemia and diabetes mellitus were recorded. Hypertension was defined as being on anti-hypertensive drugs or having blood pressure greater than 140mmHg systolic or 90mmHg diastolic. Hypercholesterolemia was defined as being on cholesterol lowering therapy or a serum cholesterol > 5.2mmol/l. Diabetes mellitus was defined as a clinical diagnosis of the disease.

### Neuropsychological assessment

Cognition was assessed using the Brief Memory and Executive Test (BMET: www.bmet.info) [20, 24], which has been previously used in studies with SVD [11, 25–27]. The BMET consists of eight tasks divided into two main categories for sub-scaling, namely: 1. *executive functioning/processing speed* which includes letter-number matching, motor-sequencing, letter-sequencing, and number-letter sequencing; and 2. *memory/orientation* which includes five-item repetition, recall, and recognition memory, and basic time and place orientation questions. A full description of the tasks can be found in previously reported studies [20, 24].

The BMET has an aggregate overall measure, termed the cognitive index score, with a previously defined clinical cut-off score of ≤ 13 [20]. For the executive functioning/processing speed and memory/orientation subscales, the scores are considered parametrically. Performance across all test measures was made comparable by converting the raw-scores into z-scores based on the control mean and standard deviations stratified by age band. Compound scores for the two main subdomains were then calculated by averaging the relevant z-scores. The test scores of all sequencing task measures were inverted such that higher z-scores indicate better performance. Glass’ delta was used to compute effect sizes, and defined cognitive impairment as a score below a cut-off of 1.5 standard deviations (SDs) below the standardized mean of the control data [28].

Premorbid IQ was assessed by use of the revised National Adult Reading Test (NART-R) applying the UK re-standardization by Nelson and Willison [29].

### Apathy and depression

All participants completed the Geriatric Depression Scale (GDS), a thirty item self-report screening scale for depression [30]. Past research has shown that the GDS has good internal consistency (α = .86) [30], and a sensitivity of approximately 80% when compared to clinical diagnoses of depression [31]. Participants were asked to answer yes/no to questions regarding depressive symptoms. The apathy and depression questions from the scale were separated based on a model that has been previously used, deriving separate scales [11]. The apathy scale had a range of 0–6 and consisted of the following six items: ‘prefer to stay home’, ‘avoid social gatherings’, ‘dropped activities and interests’, ‘find life very exciting’, ‘hard to start new projects’ and ‘full of energy’. The depression scale, which included the remaining 24 items, had a range of 0–24. Glass’ delta was used to compute effect sizes, and defined apathy and depression as having a score 1.5 SDs below the mean of the control data. To confirm the validity of the apathy and depression subscale in this sample, we adopted a confirmatory factor analysis approach conducted in MPLUS. The structure of the apathy/depression scales was confirmed, with adequate model fit statistics (*χ*^*2*^(61) = 87.034, comparative fit index (CFI) = .979, root mean square error of approximation (RMSEA) = .047).

### Statistical analyses

Differences between participants with SVD and controls on descriptive variables and clinical parameters were analyzed using univariate ANOVA and χ^2^ tests. All analyses were corrected for age, gender and premorbid IQ.

A model of apathetic and depressive symptoms, and executive function/processing speed and memory/orientation functions in the SVD patient group was initially evaluated using structural equation modelling (SEM) path analyses in AMOS v.23 (http://amosdevelopment.com). The advantage of this technique is that it allows examination of the relationships of apathetic and depressive symptoms to cognition whilst taking their covariance into account. It also allowed us to model the impact of executive function/processing speed on memory/orientation in this group. The variance in all core variables (apathetic and depressive symptoms, executive functioning/ processing speed, memory/orientation) accounted for by age and premorbid IQ, was controlled for in the models. Missing data were examined and found to be few and sporadic (NART: n = 3). Therefore these data were imputed using an expectation maximization algorithm, as part of SPSS’s Missing Value Analysis Tool.

Model parameters were estimated using maximum likelihood estimation. To evaluate model fit we applied multiple criteria as recommended by Kline [32]: 1) The CFI where higher values indicate larger improvement over a null model (>.90 indicates a good fit); 2) The RMSEA measures the fit of the specified model to the sample covariance matrix. RMSEA < .05 indicates a good fitting model. PCLOSE gives a probability value for H_0_ RMSEA ≤.05, therefore a p-value greater than .05 indicates a good fit; 3) Chi-squared test for lack of fit (non-significant results indicate a good fit). Chi-squared difference was calculated to look for a significant change in fit from model 1 to model 2 and from model 1 to model 3. Bootstrapping (2000 samples) was also employed to estimate indirect effects in the model.

A binary logistic regression was used to determine whether or not clinically relevant apathy, depression, or cognitive deficits were co-related. Here, patients with clinically relevant increments in apathy and depression or impairments on the cognitive indices were defined based on scores ≥ 1.5 SD deviation from the control population values [33]. Then for each index of cognition a binary logistic regression was used to examine prediction of impairment based on apathy and depression. Also accounted for in the model were age, gender and premorbid IQ. The same analyses were done with additional correction for the degree of WMH. We applied additional Bonferroni correction to correct for multiple comparisons. These analyses were performed using IBM SPSS Statistics version 23.

## Results

### Descriptive statistics

Table 1 presents the demographic and clinical characteristics of both groups. Two participants over 90 years and two participants under 40 years were not included in this analysis due to lack of normative data for these age decades, leaving 196 SVD cases and 300 controls. The two groups did not differ significantly in age and years of education. There were more male participants in the SVD group and the controls had a slightly higher premorbid IQ. Missing data for key variables were as follows: years of education, SVD = 4; body mass index, SVD = 9, controls = 1; alcohol units per week, SVD = 3.

**Table 1:** Demographic and clinical variables for participants with SVD and controls.

	**SVD**	**Controls**	**p-values**
	(n = 196)	(n = 300)	
**Age**	63.51 (9.91)	62.55 (13.78)	p = .401
**Male Gender**	133 (67.9%)	138 (46%)	p = .000
**Premorbid IQ**	114.64 (9.74)	117.34 (7.05)	p = .000
**Years of education**	13.61 (3.80)	14.01 (2.80)	p = .184
**Fazekas scale WMH ≥ 2**	74 (37.8%)		
**Hypertension**	145 (74.0%)	83 (27.7%)	p = .000
**Hyperlipidaemia**	154 (78.6%)	72 (24.0%)	p = .000
**Diabetes**	45 (23.0%)	18 (6.0%)	p = .000
**Smoking** (ever)	111 (56.6%)	127 (42.3%)	p = .000
**Alcohol intake** (units/week)	8.70 (14.69)	11.57 (12.94)	p = .023
**Body mass index** (kg/m^2^)	28.26 (5.37)	25.92 (5.11)	p = .000
**Apathy**	54 (27.6%)	32 (10.7%)	p = .000
**Depression**	25 (12.8%)	29 (9.7%)	p = .175
**Total Cognitive Index**	13.59 (2.86)	15.15 (1.56)	p = .000
**Memory & Orientation**	6.74 (1.56)	7.49 (1.01)	p = .000
**Executive Functioning & Processing Speed**	6.84 (1.85)	7.66 (.93)	p = .000

Values presented are mean (SD) or number (%). Statistical tests presented are univariate ANOVA for continuous variables and χ^2^ tests for categorical variables. SVD–cerebral Small Vessel Disease; BMET–Brief Memory and Executive Test.

On Fazekas scale grading, 93 patients (47.4%) with SVD had no WMH, 29 patients (14.8%) had punctuate WMH, 30 patients (15.3%) had beginning confluence of WMH, and 44 patients (22.4%) had large confluent areas of WMH.

### Prevalence of apathy and depression and cognitive impairment

Thirty-one patients with SVD (15.8%) met the criteria for apathy only, 23 participants (11.8%) met the criteria for both apathy and depression, and 2 participants (1.0%) met the criteria for depression only. Apathy was more common in SVD cases than controls (SVD: 27.6% vs. controls: 10.7%, p = .0001) but there was no significant difference in the prevalence of depression (SVD: 12.8% vs. controls: 9.7%, p = .175).

The degree of WMH was not related to the magnitude of apathetic symptoms (F(3,196) = .984, p = .401), the magnitude of depressive symptoms (F(3,196) = .431, p = .731), presence of apathy (p = .3017) or presence of depression (p = .241).

The SVD group performed worse than the control group on the Total Cognitive Index score, and the two sub-indices, executive functioning/processing speed and memory/orientation (Table 1).

### Path analysis of the association between apathy, depression and cognition

**Fig 1:**
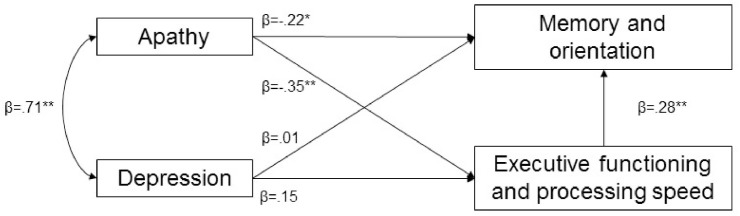
Path analysis model. Path analysis model showing that the magnitude of apathetic symptoms, but not depressive symptoms, is associated with memory/orientation and executive functioning/ processing speed. Premorbid IQ and age were both regressed into the model (not displayed). There was a strong covariance between age and depressive symptoms (*β* = -.30, *p <* .001), and between premorbid IQ and executive function/processing speed (*β* = .35, *p <* .001) and memory/orientation (*β* = .16, *p =* .019). * p < .05; ** p < .005.

The path analysis model is shown in Fig 1. Examination of the individual paths within the model indicated that apathetic symptoms were related to both the executive functioning/processing speed (*β* = -.353, *p* < .001) and memory/orientation indices (*β* = -.221, *p* = .019). Depressive symptoms, however, were not related to executive functioning/processing speed (*β* = .149, *p =* .118), nor memory/orientation (*β* = .008, *p* = .934). Bootstrap analyses showed a significant indirect effect of apathy on memory/orientation via executive function/processing speed (*β* = -.080; 95% CI:-.158, -.034, *p* < .001) There was a strong covariance between apathetic and depressive symptoms (*β* = -.718, *p* < .001). The model was well supported by fit statistics (*χ*^*2*^(1) = 1.846, *p =* .174; CFI = .996; RMSEA = .066, PCLOSE = .274).

### Associations with the presence of apathy and depression with cognitive indices

Binary logistic regression (Table 2) revealed that the presence of apathy (OR: 2.791, *p =* .014, CI: 1.323–6.325), but not the presence of depression (OR: .905, *p =* .858, CI: .301–2.717), was inversely related to the total cognitive index. When cognition was split into separate indices, the presence of apathy (OR: 2.371, *p =* .041, CI: 1.033–5.439) was related to impaired executive functioning/processing speed, but not to memory/orientation (OR: 1.505, *p =* .394, CI: .588–3.853). The presence of depression was not related to impaired executive functioning/ processing speed (OR: 1.249, *p =* .692, CI: .415–3.762) or to memory/orientation (OR: 1.631, *p =* .426, CI: .489–5.446). Additional correction for the degree of WMH did not change these results (S1 Table). After correcting for multiple comparisons by using Bonferroni correction, the significance level was at .0167. This did not change the association between presence of apathy and impaired total cognitive index; the association between the presence of apathy and impaired executive functioning/processing speed was no longer significant.

**Table 2:** Binary logistic regression analysis.

	**Impaired Cognitive Index**	**Impaired Memory/ Orientation**	**Impaired Executive function/ processing speed**
**Presence of Apathy**	OR = 2.791 (1.323–6.325)	OR = 1.505 (.588–3.853)	OR = 2.371 (1.033–5.439)
	p = .014	p = .394	p = .042
**Presence of Depression**	OR = .905 (.301–2.717)	OR = 1.631 (.489–5.446)	OR = 1.249 (.415–3.762)
	p = .858	p = .426	p = .692
**Age**	OR = .988 (.9555–1.021)	OR = .989 (.951–1.030)	OR = .978 (.944–1.013)
	p = .466	p = .602	p = .215
**Gender**	OR = 1.034 (.504–2.123)	OR = 1.412 (.616–3.233)	OR = 1.166 (.553–2.459)
	p = .927	p = .415	p = .686
**Premorbid IQ**	OR = .933 (.901-.965)	OR = .905 (.911-.981)	OR = .934 (.903-.967)
	p = .000	p = .000	p = .000

Values presented are Odd’s Ratios (95% confidence interval).

## Discussion

Our results suggest that apathy, and not depression, may be the predominant neuropsychiatric symptom associated with SVD. We found that apathy is highly prevalent in SVD, even in absence of depression, while the prevalence of depression itself was not significantly more common than in a similarly aged stroke-free control group. Apathy was unrelated to the degree of WMH in this study, indicating that apathy is present in SVD across patients with varying degrees of WMH. Furthermore the presence of apathy was associated with the extent of cognitive impairment, and specifically the impairment in executive dysfunction/processing speed. This implies that apathy may share underlying mechanisms with executive dysfunction and impaired processing speed which are the characteristic cognitive impairments seen in SVD.

We showed a dissociation between apathy and depression in patients with SVD and confirmed this by use of a confirmatory factor analysis. These findings are consistent with a previous study in a different cohort of patients with SVD, who had more severe disease with confluent WMH in addition to symptomatic lacunar stroke [11]. According to our criteria, 16% of patients with SVD had apathy but not depression, while 12% had both apathy and depression. Surprisingly only 1% of the patients had depression only. While depression is still an important neuropsychiatric comorbidity of SVD, the current data suggest that apathy may be the main underlying non-cognitive psychological disorder. Many depression scales include both apathetic and depressive symptoms, and it may be the failure to differentiate these that has led to an over-diagnosis of depression in SVD and an under-diagnosis of apathy.

Our binary logistic regression analysis revealed that the presence of apathy, but not depression, was associated with impaired cognitive index. After splitting global cognition into separate indices, we observed an association between the presence of apathy and executive dysfunction/processing speed, but not with memory/orientation. However, this association between apathy and impaired executive functioning/processing speed became non-significant after applying a Bonferroni correction; this may be due to the sample size. It has been suggested that dysfunction of the fronto-subcortical system, which is involved in executive functioning, is implicated in the causation of apathy [18], and that white matter damage to the cortico-subcortical pathways which connect brain regions important for regulating emotions, may cause an increase in the prevalence of apathy and depression in SVD [34]. Most recently, apathy has been associated with damage to white matter tracts connecting regions in the frontal lobe with both subcortical structures and the temporal lobe [11], even when controlling for cognitive functioning. Some regions associated with apathy overlap with those associated with cognitive functioning, especially processing speed [35]. This may imply that apathy and executive function share some underlying mechanisms.

In the current study, the magnitude of apathetic symptoms was directly inversely associated with memory/orientation, and indirectly via executive function/processing speed; in contrast the presence of apathy (using a predefined cut-off score to define the condition), was not associated with memory/orientation. This apparent discrepancy could be explained by the relatively small percentage of patients with memory deficits, and by attention being needed to be able to encode and process information in the working memory [36], but also because the neurocognitive systems supporting memory might have less overlap than those that modulate motivation. Previous studies have demonstrated that episodic memory is relatively spared in early stages of SVD [3, 7, 20].

In this study we recruited unselected non-demented patients presenting with lacunar stroke who had varying degrees of WMH from none to severe confluent changes. This represents a broader spectrum than a previous study showing dissociation between apathy and depression in SVD which only included patients with lacunar stroke and confluent WMH [11]. SVD represents a spectrum of disease from asymptomatic WMH in community populations, through lacunar stroke to patients presenting with cognitive impairment and dementia [37]. Further studies are required to determine whether these findings apply to the full spectrum of SVD including patients with apparently “asymptomatic” WMH, enlarged perivascular space, cerebral microbleeds and brain atrophy.

We derived apathy and depression scores from the Geriatric Depression Scale. It has been previously shown that it is possible to dissociate these two behavioral impairments using this approach [11]. The cut-offs for defining the presence of apathy and depression were then derived on the mean and standard deviation values in controls. Future studies in this area, however, would benefit from a more extensive detailed assessment of apathy to replicate the current results. For instance, by using the apathy evaluation scale [38] which divides apathy into behavioral, cognitive and emotional symptoms, or the Lille Apathy Rating Scale [39], dividing apathy into subdomains enabling the development of patient specific profiles. Furthermore future studies should use a more comprehensive cognitive test battery to explore the relationship between apathy and cognition in more detail.

Using clinical cut-offs for apathy and depression in this study resulted in a small number of patients with apathy and especially depression, and therefore we could not compare patients with depression only with the other patient groups. In contrast to this study, previous studies have found that both presence of apathy and depression were increased in patients with SVD [12, 40]. This could be due to the fact that previous studies have used scores such as the total GDS score to define depression which include both apathetic and depressive symptoms. When we split them as in this paper we find differential associations. Performing a path analysis using continuous scores of the magnitude of apathetic and depressive symptoms demonstrates an association between apathetic symptoms and cognition. Due to the cross-sectional nature of this study, interpretation of the results in terms of causality must be done cautiously. Follow-up studies should examine the course and interaction of apathy, depression and cognition in SVD.

Apathy is defined as a diminished motivation, and lack of effort and executive control [13–15], which could result in loss of interest and effort in cognitive testing. Hence, the relation between apathy and cognitive impairment could be due to an incapability of goal-oriented thinking and behavior, instead of common underlying brain damage. However, in this study, participants were sufficiently motivated to overcome apathetic symptoms to take part, and we found an association only between apathy and impaired executive functioning/processing speed. A lack of effort in our participants would have also led to a poor performance in memory/orientation where we found no significant relationship with the apathy score when we used cut-off scores for cognitive impairment on each index score. Future studies should include objective measures of effort, for example by adding tasks to investigate physical effort [41].

In another study, apathetic patients were found to be older than non-apathetic patients post-stroke [40]. In this SVD population, age was associated with the magnitude of depressive symptoms, but not with the magnitude of apathetic symptoms. Given the small number of patients with apathetic and depressive symptoms, we were not able to explore possible variation by age and gender in this study population. However, all analyses were corrected for age and gender, and the associations between apathy and cognition remained significant after these adjustments. Nevertheless, future studies should address possible differences by age and gender in SVD.

This study has a number of clinical implications. Apathy may have a major impact on quality of life of both the patient and their partner/carer and may impact on rehabilitation after stroke. The high prevalence of apathy in SVD, which is often undetected, emphasizes that it is important to screen for apathetic symptoms in addition to screening for depressive symptoms to guarantee an optimal treatment for the patient. However, how to treat apathy in SVD best remains to be determined. More data is needed on the effectiveness of antidepressant and cognitive behavioral therapy on improving apathetic, rather than depressive, symptoms in SVD. In other neurological disorders such as Alzheimer’s or Parkinson’s disease, pharmacological and cognitive behavioral psychotherapy approaches for apathy have been explored. Recently, dopaminergic agonists have been suggested as treatment of apathy [18, 42]. Future studies should explore the effectiveness of these treatment options in SVD.

## Supporting information

S1 Table Binary logistic regression analysis with additional correction for WMH. (DOCX)

S1 File Dataset. (XLS)
