# Supplementary material for: Apathy, but not depression, is associated with executive dysfunction in cerebral small vessel disease
Source: PLoS One. 2017 May 11;12(5):e0176943. doi: 10.1371/journal.pone.0176943 (PMC5426624; doi:10.1371/journal.pone.0176943)
Supplement: S1 Table — (DOCX) [file pone.0176943.s001.docx]

**S1 Table.** **Binary Logistic Regression Analysis with additional correction for WMH.**

|  | Impaired Cognitive Index | Impaired Memory/ Orientation | Impaired Executive function/ processing speed |
| --- | --- | --- | --- |
| Presence of Apathy | OR=2.822 (1.217-6.546) | OR=1.471 (.558-3.878) | OR=2.399 (1.021-5.639) |
|  | p=.016 | p=.435 | p=.042 |
| Presence of Depression | OR=.856 (.278-2.631) | OR=1.629 (.478-5.553) | OR=1.230 (.398-3.800) |
|  | p=.786 | p=.436 | p=.719 |
| Age | OR=.987 (.951-1.023) | OR=.577 (.946-1.032) | OR=.978 (.941-1.016) |
|  | p=.474 | p=.577 | p=.248 |
| Gender | OR=1.046 (.504-2.174) | OR=1.472 (.636-3.406) | OR=1.149 (.539-2.450) |
|  | p=.903 | p=.366 | p=.719 |
| Premorbid IQ | OR=.932 (.900-.964) | OR=.946 (.911-.982) | OR=.933 (.901-.966) |
|  | p=.000 | p=.004 | p=.000 |
| WMH |  |  |  |
| Punctuate  WMH | OR=.738 (.265-2.057) | OR=.738 (.211-2.584) | OR=.995 (.353-2.805) |
|  | p=.561 | p=.634 | p=.992 |
| Beginning  confluence of  WMH | OR=.713 (.266-1.913) | OR=1.502 (.525-4.297) | OR=.692 (.246-1.946) |
|  | p=.502 | p=.448 | p=.485 |
| Large  confluence of  WMH | OR=1.131 (.457-2.800) | OR=.960 (.324-2.851) | OR=1.121 (.434-2.890) |
|  | p=.790 | p=.942 | p=.814 |

Values presented are Odd’s Ratios (95% confidence interval).
